# Ferulic acid lipid nano capsules versus its native form in alleviating diabetic nephropathy induced in rats through TGF-β1/Hippo pathway crosstalk modulation

**DOI:** 10.1038/s41598-024-81175-7

**Published:** 2025-03-31

**Authors:** Mona H. Zohny, Yousra M. El-Far, Mohamed Fawzi Kabil, Sahar E. El-Swefy, Ibrahim M. El-Sherbiny, Mamdouh M. El-Shishtawy

**Affiliations:** 1 Department of Pharmacology and Biochemistry, Faculty of Pharmacy, Delta University for Science and Technology, Gamasa, 11152 Egypt; 2 Department of Biochemistry, Faculty of Pharmacy, Mansoura University, Mansoura, 35516 Egypt; 3 Nanomedicine Research Labs, Center for Materials Sciences, Zewail City of Science and Technology, 6th of October City, Giza 12578 Egypt

**Keywords:** Ferulic acid, Ferulic acid lipid nano-capsules, Diabetic nephropathy, Hippo pathway, Transforming growth factor-β1., Biochemistry, Chemical biology, Drug discovery

## Abstract

Diabetic nephropathy is one of the most common leading causes of end-stage renal disease with multifactorial pathophysiological mechanisms. TGF-β1 and Hippo pathway have been reported to have significant role in different kidney diseases. In addition, ferulic acid (FA) has been proposed to have pharmacological actions in different disorders such as Alzheimer, diabetes mellitus, kidney, and cardiovascular diseases but with limited oral use due to poor absorbance and bioavailability. So, recent trends aim to include FA in nano-formulations to improve its absorbance and bioavailability and to make best use of its pharmacological actions when administered orally. Thirty Sprague Dawley male rats were divided into five groups (*n* = 6). After 28 days, rats were sacrificed, serum and kidney tissue were isolated, histopathological examination, serum creatinine level and oxidative status biomarkers in kidney tissue were estimated, besides ELISA measurements of TGF-β1, PTEN, COX2, and GLUT3 and the relative gene expressions of *MST1* and *TEAD4* by qRT-PCR. Treated groups show improvement of the investigated parameters in variable degrees. Noteworthy, FA nano-formulation shows superior action over double of the native form.

**Supplementary Information:**

The online version contains supplementary material available at 10.1038/s41598-024-81175-7.

## Introduction

Diabetic nephropathy (DN) is a consequence of long-term hyperglycemia and high blood pressure making it one of the most common complications in diabetic patients. It is considered a heavy burden to society as it is the chief cause of end-stage renal disease and cardiovascular morbidity and mortality in these patients^1^. Wide variety of molecular mechanisms contributes to the pathogenesis of DN making it complicated and multifactorial. Conventionally, these mechanisms included metabolic and hemodynamic factors including prolonged hyperglycemia, reactive oxygen species (ROS), advanced glycated end-products, protein kinase C and the renin-angiotensin-aldosterone system (RAAS)^2^. However, the main management protocols of DN, including modulation of blood glucose level and RAAS inhibitors, are found to be inefficient where patients still unavoidably reach end-stage renal disease^3^. Hence, understanding the cascade that triggers this pathogenesis, designing innovative management strategies and introducing new therapeutic targets beyond hyperglycemia and hypertension are crucial to achieve better prognosis of DN.

The Hippo pathway is a signaling pathway that was first discovered and identified in *Drosophila fly.* It is found to have evolutionary roles in cell growth, epithelial homeostasis, tissue proliferation, immune modulation, and apoptosis^4^. The core of the Hippo pathway in mammals consists of mammalian sterile 20-like kinase (MST) 1/2 and large tumor suppressor (LATS) 1/2 as a kinase cascade, in addition to the transcriptional co-activator yes associated protein-1 (YAP1) and the transcriptional co-activator with PDZ-binding motif (TAZ) as downstream effectors^5^. In case of pathway inhibition, the dephosphorylated co-activators YAP/TAZ translocate to the nucleus, interact with transcriptional enhanced associate domain (TEAD) transcription factor family resulting in expression of number of genes^6^. Besides being involved in various human cancers, fibrotic diseases, pulmonary, cardiovascular and neuronal disorders^7–11^, dysregulation of the Hippo pathway has been recently spotted under diabetic conditions and kidney diseases^12–15^. On the other hand, its role in DN has not been fully explained to date, so understanding its molecular functions and recognizing potential therapeutic targets has come to forefront of its research field.

Likewise, transforming growth factor-β1 (TGF-β1), a pleiotropic cytokine produced by different types of cells, is found to be implicated in plentiful biological processes such as differentiation, tumor suppression and metastasis, cell division, immunity, and apoptosis^16^. Noteworthy, previous studies suggested that TGF-β1 signaling plays a critical pathophysiological role in DN leading to functional and morphological changes in kidneys of diabetic patients^17^.

Of interest, treatments with antioxidant, anti-inflammatory and anti-cancer effects were found to have promising therapeutic actions on DN^18,19^. Ferulic acid (FA), a phenolic acid commonly found in the fruits, seeds, and leaves of most plants, is found to induce significant antioxidant^20^, antiviral^21^, anti-inflammatory^22^, anti-allergic^23^, anti-microbial^24^, hepato-protective^25^ and anti-carcinogenic effects^26^. Moreover, foods enriched with FA are found to decrease amyloid pathogenesis in Alzheimer disease through management of oxidative status and inflammation which are considered as significant therapeutic targets of DN^27^. Unfortunately, oral use of FA has limitations due to its pharmacokinetics as it has marked first-pass effects limiting its bioavailability. Also, the anionic form of FA at physiological pH is three orders of magnitude larger than the neutral one making it incapable to cross membranes^28^. Therefore, a good strategy to increase the bioavailability of FA is to incorporate it in nano-formulations in order to make best use of its pharmacological effects and increase its oral bioavailability and absorbance.

Experimental DN models in rats are mostly used to explore its pathological mechanisms and to pinpoint suitable possible therapeutic targets. Streptozotocin (STZ) a selective beta cells destructing agent develops one of the best characterized DN models on account of its reproducibility, ease of induction and representing massive characteristics of its pathogenesis^29^. Therefore, the current study is designed to investigate and compare the possible actions of FA and ferulic acid lipid nano-capsules (FA-LNCs) on DN experimental model in the context of TGF-β1/Hippo pathway crosstalk and modulation.

## Materials and methods

Ferulic acid (99% purity) was purchased from Sigma-Aldrich St. Louis, MO (USA). Lipoid® S75-3 (Soybean lecithin with 69% of phosphatidylcholine was provided as a gift from Lipoid GmbH (Ludwigshafen, Germany), and Cremophor® RH 40 was purchased from BASF (Germany).

### Preparation of FA suspension

Ferulic acid was suspended in 0.5% carboxy methyl cellulose (CMC)^26^ to the desired concentrations. FA (25 mg/kg/day)^30^, and FA (50 mg/kg/day)^31^ are in the range of those used in other studies applied for the same animal species and the same treatment period^32^.

### Preparation and assessment of ferulic acid lipid nano-capsules with dose of 25 mg/kg/day (FA-LNCs25)

#### Preparation of FA-LNCs25

Ferulic acid lipid nano-capsules 25 (FA-LNCs25) were prepared as described by Heurtault et al.^33–36^. In summary, FA (50 mg), Labrafac Lipophile oil (10% w/w), Lipoid® S75 as a lipophilic surfactant (1.5% w/w), Cremophor® RH 40 (10% w/w) and water (30% w/w) were added and stirred using magnetic stirring. The previous mixture was subjected to three heating-cooling cycles from 70 °C to 90 °C in order to form water in oil (w/o) emulsion. To reach the desired concentration, water (4 °C, 50% v/v) for dilution is added by mixing for 5 min to allow the irreversible shock and form the w/o emulsion.

#### Assessment of FA-LNCs25

##### Determination of particle size, polydispersity index and zeta potential

The lipid nano-capsules were diluted with ratio of 1:100 by phosphate buffer saline pH 7.4 before the measurements of particle size (PS), polydispersity index (PDI) and zeta potential (ZP) *via* the aid of dynamic light scattering (DLS) on a Zeta sizer Nano series (Malvern Instruments SA, Worcestershire, UK)^37^.

##### Surface morphology

The morphological examination of the FA-LNCs was evaluated by high resolution transmission electron microscope (HR-TEM) (model JEM- 100 S, Joel, Tokyo, Japan)^38^.

##### In-vitro release of FA from FA-LNCs25

The release of FA from FA-LNCs25 was studied using dialysis bag method as described in^33–38^. The release media was phosphate buffer saline (20 ml, pH 7.4) using 10% methanol to achieve the sink conditions. Also, the quantification of the FA in the withdrawn samples was done using UV spectrophotometer at 320 nm. Each release point is a mean of triplicate measurements in addition to a statistical calculation of standard deviation represented as the error bars.

##### Assessment of the storage stability

The stability of the FA-LNCs25 was evaluated in accordance with the significant change in PS and PDI over 3-months at 4–8 °C^38,39^.

### Experimental study and treatment protocol

#### Animals

Experiments were performed on 60 adult male Sprague Dawley rats. Rats were purchased from Theodor Bilharz Research Institute, Cairo (Egypt), with body weight of 200 ± 20 g. They were kept in metabolic steel mesh cages (3 rats per cage). Rats were fed standard pellet chow (El-Nasr Chemical Company, Cairo, Egypt), allowed free access to tap water, and kept under the same circumstances for 7 days before the experiment for acclimatization. The experimental protocols were approved by Faculty of Pharmacy, Mansoura University, Egypt by the licensing committee (Research Ethics Committee) (Code No. 2023-53, Date of approval 20th March 2023).

All experiments were performed in accordance with relevant guidelines and regulations in accordance with The ARRIVE guidelines 2.0^40^.

##### Accordance

We confirmed that all experiments in this study were performed in accordance with the relevant guidelines and regulations.

##### Arrive

All the procedure of the study is followed by the ARRIVE guidelines.

#### Diabetic nephropathy rat model

Before starting our treatment protocols, a preliminary study was conducted. Its aim was to confirm the diabetic nephropathy induction and to ensure quantifying of renal damage.

#### Preliminary study (PS)

Twenty-four rats were injected intravenously with streptozotocin (STZ) (55 mg/kg). 48 h later, rats were provided with 15 g/L sucrose solution to prevent severe hypoglycemia upon destruction of β cells of Langerhans islets. 72 h later, rats with blood glucose level more than 250 mg/dl were considered diabetic and used for the study^41^. Rats were randomized and divided into four groups (*n* = 6 each). In addition to six rats with the same age and weight were kept under the same conditions as normal control group without injection of STZ. These rats were sacrificed after different periods of time and their kidneys were fixed in neutral-buffered formalin (El-Nasr Chemicals Co, Cairo, Egypt) for succeeding histopathological examination.

#### Main study

Twenty-four rats were subjected to the same procedure applied in the preliminary study. Rats were randomized and divided into four groups (*n* = 6 each). In addition to six rats with the same age and weight were kept under the same conditions as normal control group without injection of STZ.

**The studied groups and their administration outlines:**

**Preliminary study groups**

- **PSNC**: Normal control group.
- **PS4**: Diabetic rats sacrificed after 4 weeks of STZ injection.
- **PS6**: Diabetic rats sacrificed after 6 weeks of STZ injection.
- **PS8**: Diabetic rats sacrificed after 8 weeks of STZ injection.
- **PS10**: Diabetic rats sacrificed after 10 weeks of STZ injection.

**Main study groups**

- **Normal group.**
- **DN group** including 10 weeks’ STZ-induced diabetic rats.
- **FA25 group** was given orally 1 ml of FA (25 mg/kg/day), after 10 weeks of STZ injection and continued for 28 days.
- **FA50 group** was given orally 1 ml of FA (50 mg/kg/day), after 10 weeks of STZ injection and continued for 28 days.
- **FA-LNCs25 group** was given orally 1 ml of FA-LNCs (equivalent to 25 mg/kg/day), after 10 weeks of STZ injection and continued for 28 days.

### Collection of serum and kidney tissue

There were no surgical or post-surgical procedures. There was no induction of pain during the experiment so there was no need for pain killers, but if needed at any point the animals will be given ketoprofen (3 mg/kg/IM). At the end of the experiment, rats were prevented from food and water for 8 h to eliminate variability in the investigated parameters^42^. Animals were deeply anesthetized by secobarbital (500 mg/kg; i.p.)^43^. Through a retro-orbital vein puncture, 5 ml blood samples were withdrawn and left to clot. For serum separation, samples were centrifuged at 4000 rpm for 15 min. The serum was aliquoted and stored at −80 °C. Euthanasia was performed by neck dislocation during the action of secobarbital. Kidneys were dissected out, weighed, and divided into two sections.

- For histopathological examination, the first part was fixed in neutral-buffered formalin (El-Nasr Chemicals Co, Cairo, Egypt).
- For assessment of targeted parameters, the second part was weighed and washed with phosphate buffered saline solution, pH 7.4. The homogenization was performed with a glass homogenizer in ice-cold sodium potassium phosphate buffer (0.01 M, pH 7.4) containing 1.15% KCl. Centrifugation was accomplished for 5 min at 5000×g at 4 °C and the resulted clear solution was collected and stored at −80 °C.

Animal carcasses were disposed of in biological boxes to be buried later.

### Histopathological investigation

After 24 h, kidney samples from the neutral-buffered formalin were fixed in paraffin liquid and sliced into 4-µm sections; then stained with hematoxylin and eosin (H&E). Histopathological images and the corresponding numerical scores (Table 3) are to explain the degree of tubular cell necrosis as described formerly by Mohan et al.^44^, . Histopathological study was performed blindly by a pathologist.

### Determination of oxidative stress and antioxidant biomarkers in kidney tissue

The levels of malondialdehyde (MDA) and glutathione (GSH) were determined in kidney tissue spectrophotometrically using Biodiagnostic kits (Cairo, Egypt) according to the manufacturer’s instructions.

#### ELISA measurements of creatinine level in serum, PTEN (phosphatase and tensin homolog), TGF-β1 (transforming growth factor beta 1), COX2 (cyclooxygenase 2) and GLUT3 (glucose transporter 3) levels in kidney tissue

All measurements were performed using ELISA kits supplied by Cloud-Clone Corp USCN Life Science Inc. (Wuhan, China) according to the manufacturer’s instructions.

#### qRT-PCR for the m-RNA expressions of mammalian sterile 20 like protein kinase 1 (*MST1*) and TEA domain transcription factor 4 (*TEAD4*) in kidney tissue

RNeasy Mini kit (Qiagen, Germany) was used for total RNA extraction from kidney tissue in an RNase-free environment, following the manufacturer’s protocol. NanoDrop 2000 spectrophotometer (Thermo Scientific, USA) was used for measuring RNA amount and purity. Quantiscript reverse transcriptase (QuantiTect reverse transcription kit, Qiagen, Germany) was used for the reverse transcription of 1 µg pure RNA. Thermocycler Rotor-Gene Q (Qiagen, Hilden, Germany) and SYBR Green PCR Master Mix (Qiagen, USA) were used for the PCR reaction. qPCR was done in triplicates, including no-template controls. Primer Express 3.0 was used for gene specific PCR primer design (Applied Biosystems, USA) (Table 1). The designed primer was blasted on NCBI/Blast to ensure its specificity to the required gene. The comparative cycle threshold (Ct) (2^−∆∆Ct^) method was used for the calculation of the relative expression of the studied genes. All values were normalized to the (β-actin) gene as an invariant endogenous control in the same sample. Rotor-Gene Q Software 2.1 (Qiagen) was used for data analysis.

**Table 1:** The primer set used for the amplification of rat β-actin, MST1 and TEAD4 genes.

Gene symbol	Primer sequence from 5′–3′	ACCN
β-actin	F: TCCGTCGCCGGTCCACACCC R: TCACCAACTGGGACGATATG	NM_031144.3
MST1	F: GCTAAAGTGAAGTGGACGGATACC R: GGAACAGTTGCTACCAGAGTGTCAG	NM_001107800.1
TEAD4	F: ACAGTGACCCCTACCTCGAA R: GGTCTGCCCAGAACTTCACA	F1M665

*ACCN* The accession number of the sequence in the Entrez Nucleotide Database,* β-actin* Reference housekeeping gene, *MST1* Mammalian sterile 20-like kinase 1,* TEAD4* TEA domain transcription factor 4,* F* Forward,* R* Reverse.

### Statistical analysis

GraphPad Prism software, version 8 (GraphPad Software Inc., La Jolla, CA, USA) was used for statistical analysis. To analyze differences between groups, One-way analysis of variance (ANOVA) was used. Data are expressed as the mean ± standard deviation (SD). The threshold for statistical significance was *p* < 0.05 and *p* < 0.001.

## Results

### Assessment of ferulic acid lipid nano-capsules 25 (FA-LNCs25)

FA-LNCs25 was fabricated *via* the aid of a solvent-free phase inversion method which facilitates the preparation of small FA-LNCs25 using thermally manipulated method to convert (w/o) into oil in water (o/w) system.

#### Determination of particle size, polydispersity index and zeta potential

The obtained results as shown in Table 2 revealed that the average particle size of FA-LNCs25 was about 41.73 ± 1.41 nm. Regarding the polydispersity index (PDI) of the prepared FA-LNCs25, it was 0.1 ± 0.02 which is less than 0.4^36,37^ and indicates the homogenous distribution and dispersion of the formulation. Moreover, the surface charge of the nano-formulation was found to be negatively charged just around − 16.21 ± 0.25.

**Table 2:** Dynamic light scattering (DLS) analysis of prepared ferulic acid lipid nano-capsules 25 mg/kg/day (FA-LNCs25) at 0- and after 3-months (*n* = 3).

	Freshly prepared formulations			Formulations after 3-months	
	PS ± SD	PDI ± SD	ZP ± SD	PS ± SD	PDI ± SD
FA-LNCs25	41. 73 ± 1.41	0.1 ± 0.02	− 16.21 ± 0.25	40.23 ± 1.41	0.11 ± 0.016

*PDI* Polydispersity index, *PS* Particle size, *ZP* Zeta potential.

#### Surface morphology

**Fig. 1:**
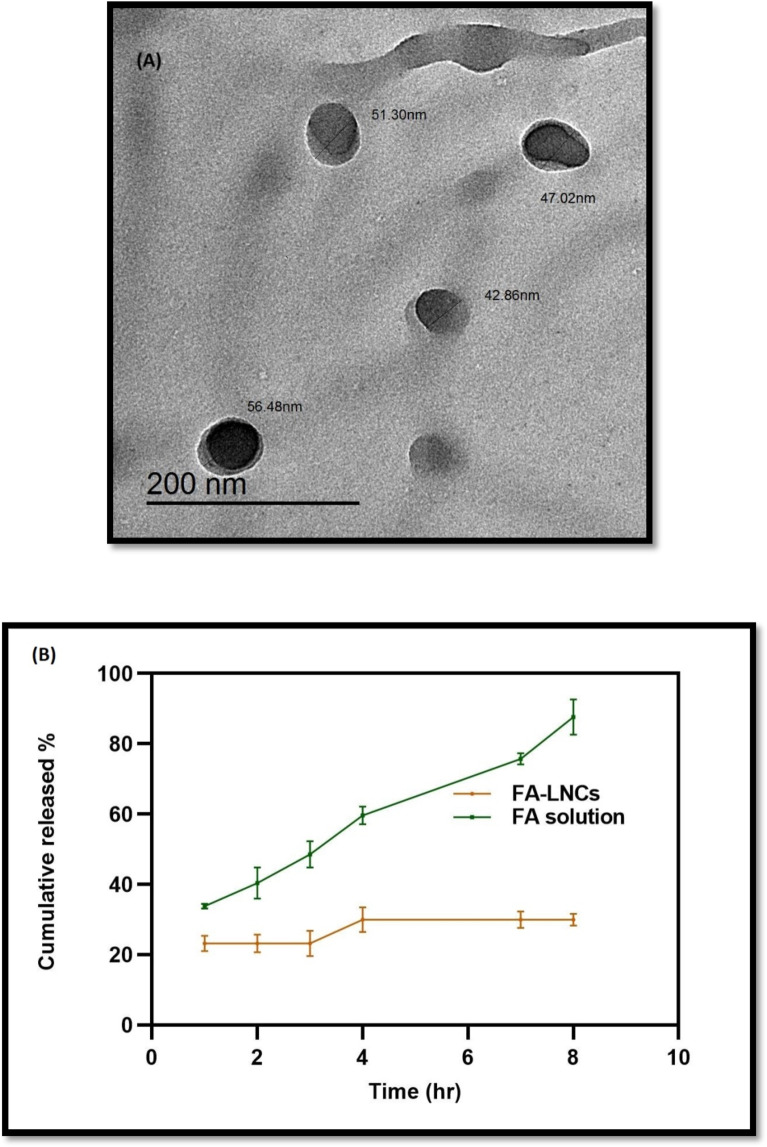
Assessment of ferulic acid lipid nano-capsules 25 mg/kg/day (FA-LNCs25). (**A**) High Resolution-Transmission electron microscopic images FA-LNCs25, magnification power X: 30,000. (**B**) The release performance of ferulic acid (FA) from ferulic acid lipid nano-capsules 25 mg/kg/day (FA-LNCs25) over a period of 8 h. Release points were represented as mean ± SD, *n* = 3.

The morphological assessment of the FA-LNCs25 appeared as spherical nano-capsules without any incidence of particle aggregation as in Fig. 1A. Moreover, the particle size of these nano-capsules agreed with the DLS measurements.

#### In-vitro release of FA from FA-LNCs25

As noted from the Fig. 1B, FA solution and FA-LNCs25 solution exerted different release profiles over the studied period of 8 h. FA solution demonstrates 80% of FA released freely from the solution during the study time which is considered as a burst drug release. On the other hand, the FA-LNCs25 demonstrates only 20% of drug release in a controlled manner including a plateau period. This is anticipated to the sustained release of the FA from the lipid nano capsules which represents a promising dosage form of the drug under investigation.

#### Assessment of the storage stability

As shown in Table 2, no significant changes in the particle size of prepared FA-LNCs25 (*p* > 0.05). Furthermore, there is no substantial increment in the PDI of the freshly prepared FA-LNCs25 and after proceeding of 3-months (*p* > 0.05); and the values of PDI after storage did not exceed the threshold mean of homogenous distribution < 0.4 which in turn illustrate the non-aggregated and the perfect stability of the prepared nano-capsules.

### Histopathological image of the preliminary study

**Fig. 2:**
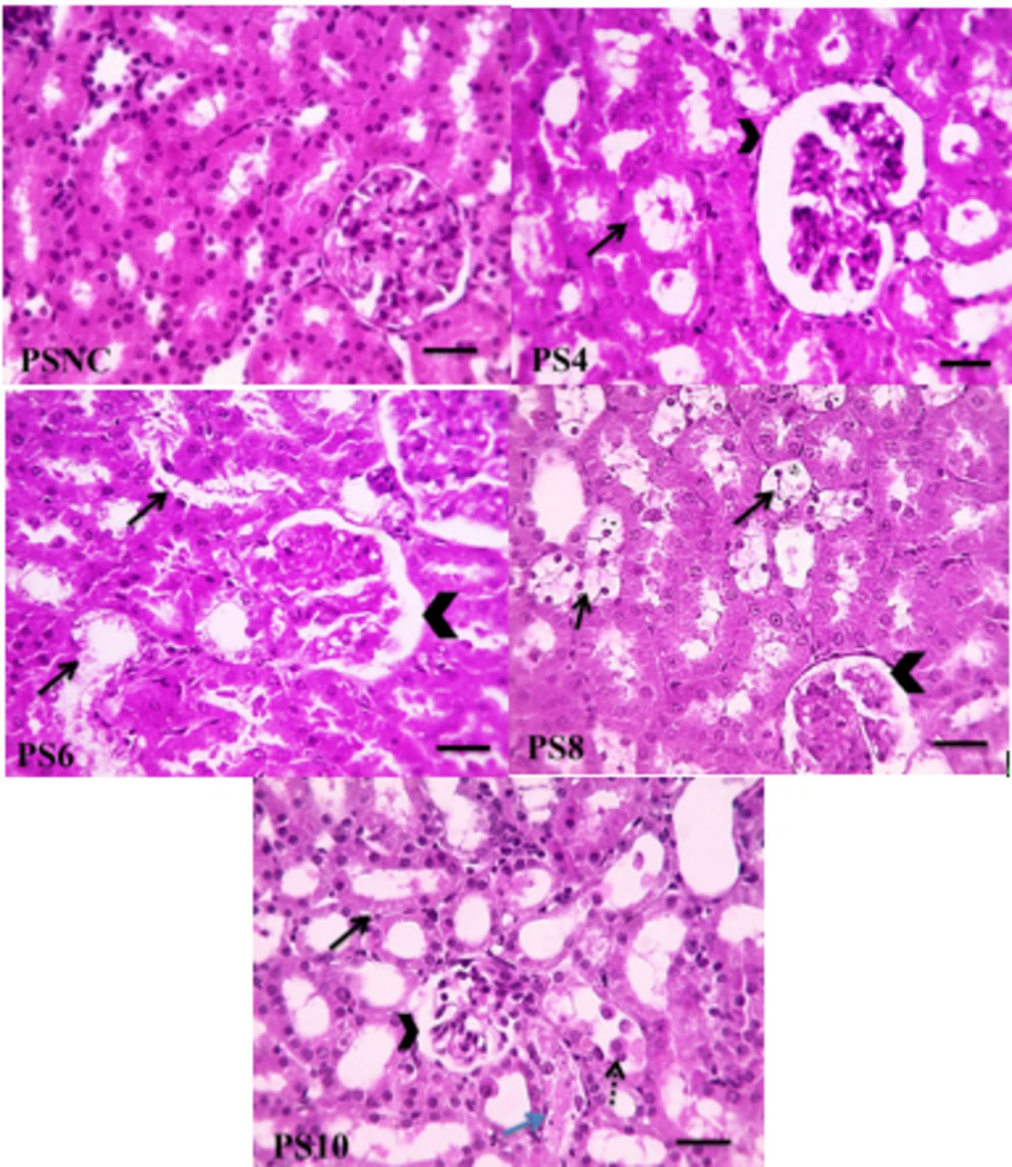
Microscopic images of H&E-stained renal cortical sections of: (PSNC) Normal control group (PSNC). (PS4) Diabetic rats sacrificed after 4 weeks group. (PS6) Diabetic rats sacrificed after 6 weeks group. (PS8) Diabetic rats sacrificed after 8 weeks group. (PS10) Diabetic rats sacrificed after 10 weeks group. X: 400 bar 50.

Figure 2A and Table 3 illustrate microscopic images of H&E-stained renal sections showing normal cortex of PSNC group with a corresponding score of 0 (Fig. S1). Figure 2B illustrates renal sections from PS4 group showing dilated Bowman’s capsule (arrowhead), tubular dilation with cast formation (black arrows) with a corresponding score of 2 (Fig. S2). Figure 2C illustrates renal sections from PS6 group showing dilated Bowman’s capsule (arrowhead), mild epithelial hydropic degeneration (black arrows) with a corresponding score of 1 (Fig. S3). Figure 2D illustrates renal sections from PS8 group demonstrating dilated Bowman’s capsule (arrowhead), moderate renal epithelial hydropic degeneration (black arrows) in some tubules with a corresponding score of 2 (Fig. S4). Figure 2E illustrates renal sections from PS10 group showing shrunken glomeruli (arrowhead), diffuse tubular dilation with epithelial degeneration (black arrows), necrosis (blue arrows) and desquamation (dashed arrows) with a corresponding score of 4 (Fig. S5).

**Table 3:** Scoring the degree of tubular necrosis in preliminary study and main study.

Preliminary study			Main study		
Group	Degree of tubular necrosis Median (Q1, Q3)	*p*-value < 0.001 Pairwise different from*	Group	Degree of tubular necrosis Median (Q1, Q3)	*p*-value < 0.001 Pairwise different from*
PSNC	0 (0, 1)	PS10	N	0 (0, 0)	DN, FA25
PS4	2 (1, 2)		DN	4 (4, 4)	N, FA-LNCs25
PS6	1 (0, 1)	PS10	FA25	3 (3, 3)	N
PS8	2 (1, 2)		FA50	2 (2, 2)	
PS10	4 (4, 4)	PSNC, PS4	FA-LNCs25	1 (1, 1)	DN

*PSNC* Normal control group, *PS4* Diabetic rats sacrificed after 4 weeks of STZ injection, *PS6* Diabetic rats sacrificed after 6 weeks of STZ injection, *PS8* Diabetic rats sacrificed after 8 weeks of STZ injection, *PS10* Diabetic rats sacrificed after 10 weeks of STZ injection, *N* Normal group, *DN* Diabetic nephropathy control group, *FA25* Effect of ferulic acid 25 mg/kg/day, *FA50* Effect of ferulic acid 50 mg/kg/day, *FA-LNCs25* Effect of ferulic acid lipid nano-capsules 25 mg/kg/day. *P-value is reported for Kruskal Wallis test. **Dunn test was used as post hoc test.

### Effect of different treatments on kidney histopathological image

**Fig. 3:**
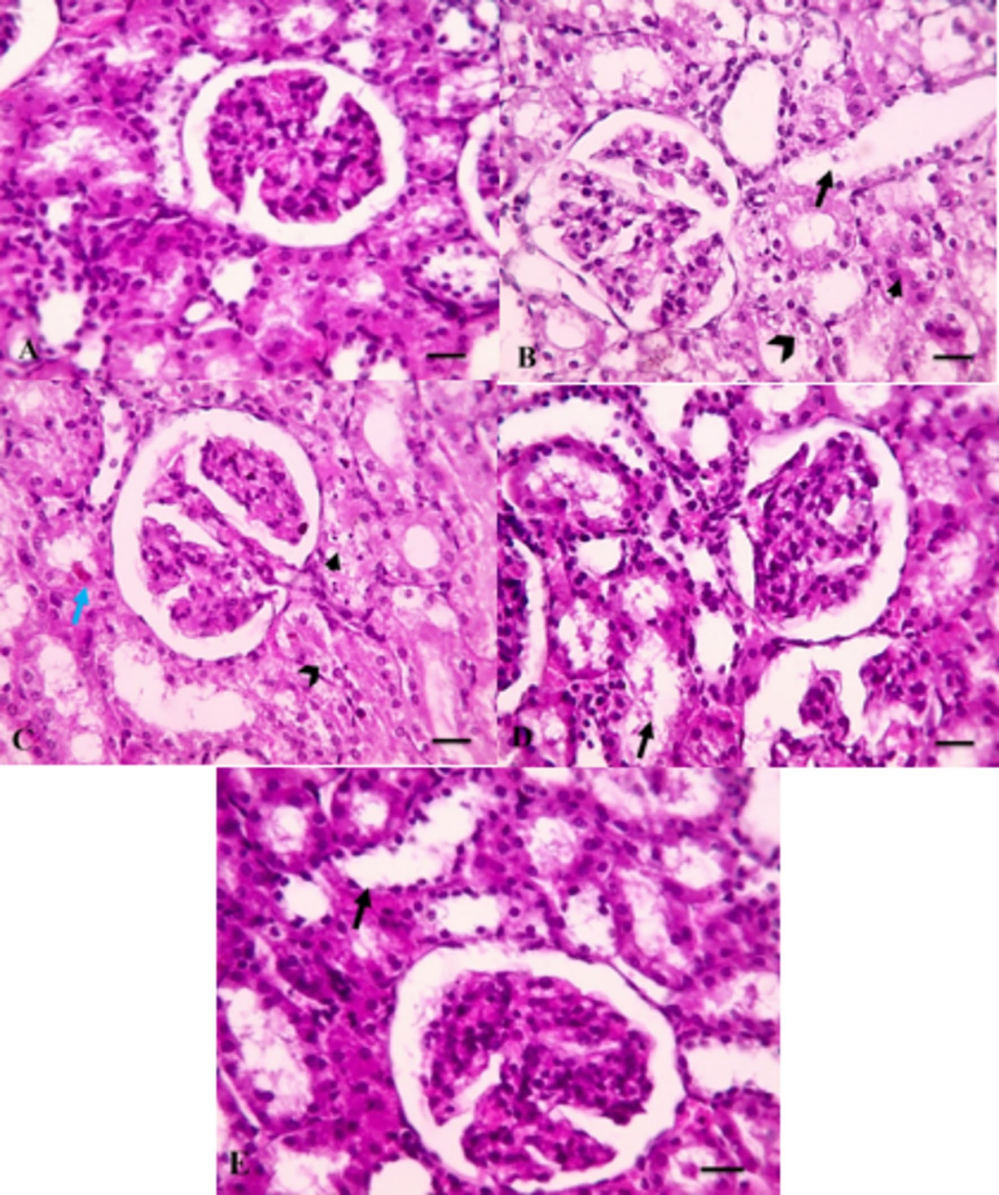
Microscopic images of H&E-stained renal cortical sections of: (**A**) Normal group. (**B**) Diabetic nephropathy control group (DN). (**C**) Effect of ferulic acid 25 mg/kg/day (FA25). (**D**) Effect of ferulic acid 50 mg/kg/day (FA50). (**E**) Effect of ferulic acid lipid nano-capsules 25 mg/kg/day (FA-LNCs25). X: 400 bar 50.

Figure 3A and Table 3 show normal glomeruli and tubules with minimal interstitial tissue in normal group with a corresponding score of 0 (Fig. S6). Figure 3B shows renal cortical sections from DN control group showing diffuse severe tubular damage including dilation (black arrows) with epithelial lining hydropic degeneration (opened black arrowheads) and coagulative necrosis (closed black arrowheads) with a corresponding score of 4 (Fig. S7). Figure 3C shows renal cortical sections from FA25 group showing tubular dilation (black arrows) with epithelial lining hydropic degeneration (black arrowheads), brown pigment infiltration (blue arrows) and coagulative necrosis (closed black arrowheads) in few tubules with a corresponding score of 3 (Fig. S8). Figure 3D shows renal cortical sections from FA50 group showing mild tubular dilation in some tubules (black arrows) with mild cast formation with a corresponding score of 2 (Fig. S9). Figure 3E shows renal cortical sections from FA-LNCs25 group showing milder tubular dilation (black arrows) with a corresponding score of 1 (Fig. S10).

### Effect of different treatments on oxidative stress and antioxidant biomarkers in kidney tissue

**Fig. 4:**
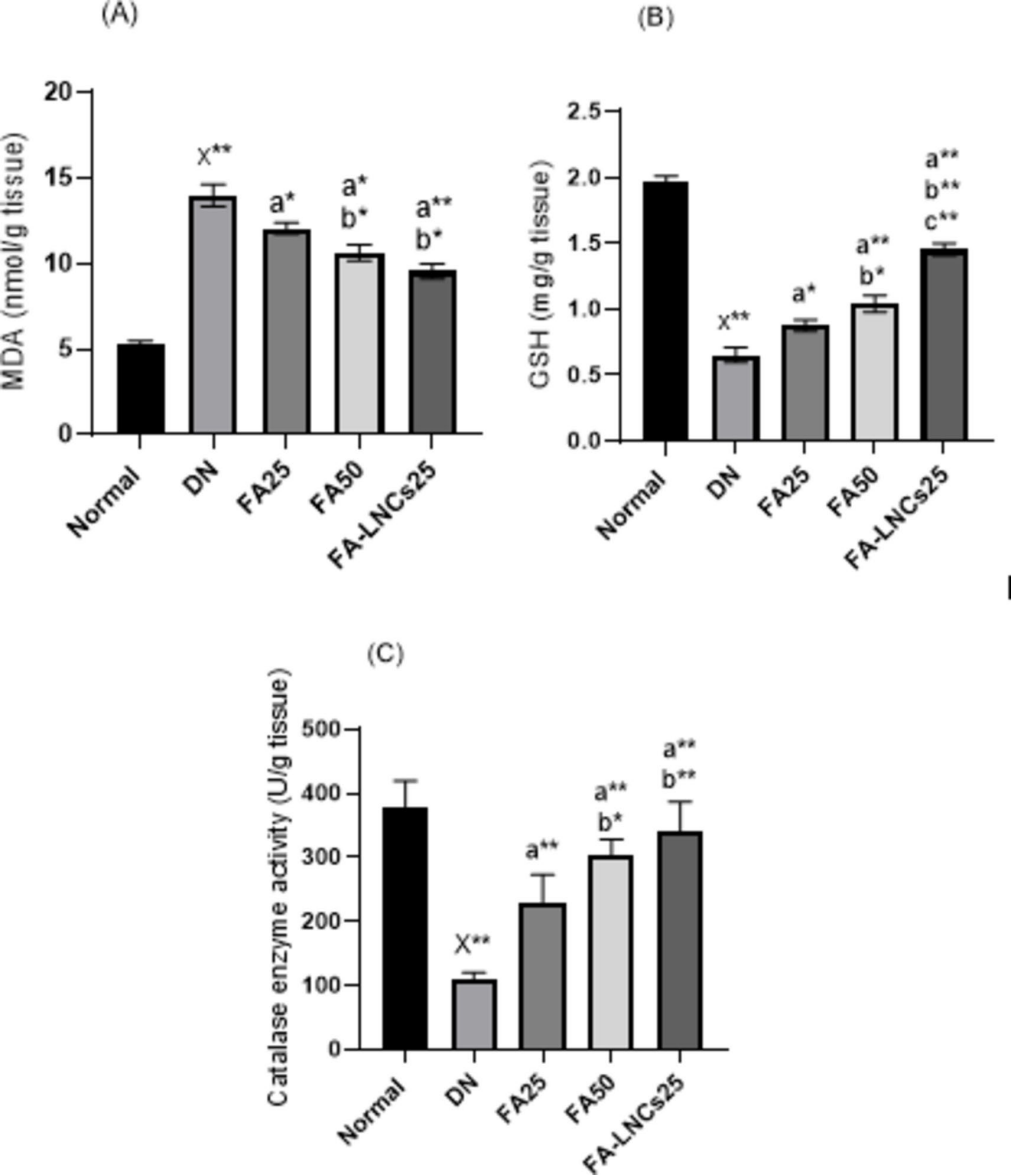
Effect of ferulic acid 25 mg/kg/day (FA25), ferulic acid 50 mg/kg/day (FA50), and ferulic acid lipid nano-capsules 25 mg/kg/day (FA-LNCs25) on kidney tissue of diabetic nephropathy model in rats. (**A**) Malondialdehyde (MDA) content. (**B**) Glutathione (GSH) content. (**C**) Catalase enzyme activity. Values were represented as mean ± SD. Number of rats in each group (*n* = 6). x: significant compared to normal group. a: significant compared to DN group. b: significant compared to FA25 group. c: significant compared to FA50 group. *Significant at *p* < 0.05 and **: significant at *p* < 0.001.

Figure 4A illustrates the effect of different treatments on malondialdehyde (MDA) as an oxidative stress biomarker in kidney tissue. A significant increase of MDA level by 62.5% (*p* < 0.001) in DN group compared to normal group is observed. This is significantly decreased in FA25, FA50 groups by 13.8% and 24%, respectively (*p* < 0.05), and in FA-LNCs25 group by 31.6% (*p* < 0.001) compared to DN group. FA50 and FA-LNCs25 groups demonstrate significant decrease in MDA level by 11.8% and 20.7%, respectively (*p* < 0.05) compared to FA25 group.

Figure 4B illustrates the effect of different treatments on glutathione (GSH) as an antioxidant biomarker in kidney tissue. A significant decrease of GSH level by 67% (*p* < 0.001) in DN group compared to normal group is detected. This is significantly elevated in FA25, FA50 and FA-LNCs25 groups by 25.3% (*p* < 0.05), 37.5% (*p* < 0.001) and 55% (*p* < 0.001), respectively, compared to DN group. FA50 and FA-LNCs25 groups show significant increase in GSH level compared to FA25 group by 16.4% (*p* < 0.05) and 40% (*p* < 0.001), respectively. Moreover, FA-LNCs25 group demonstrates significant increase in GSH level compared to FA50 group by 28.3% (*p* < 0.001).

Figure 4C illustrates the effect of different treatments on catalase enzyme activity as an antioxidant biomarker in kidney tissue. A significant decrease of catalase enzyme activity by 71.6% (*p* < 0.001) in DN group compared to normal group is detected. This is significantly elevated in FA25, FA50 and FA-LNCs25 groups by 53. 5%, 64.8% and 68. 6%, respectively (*p* < 0.001), compared to DN group. FA50 and FA-LNCs25 groups show significant increase in catalase enzyme activity compared to FA25 group by 24.4% (*p* < 0.05) and 32.7% (*p* < 0.001), respectively.

### Effect of different treatments on creatinine level in serum

**Fig. 5:**
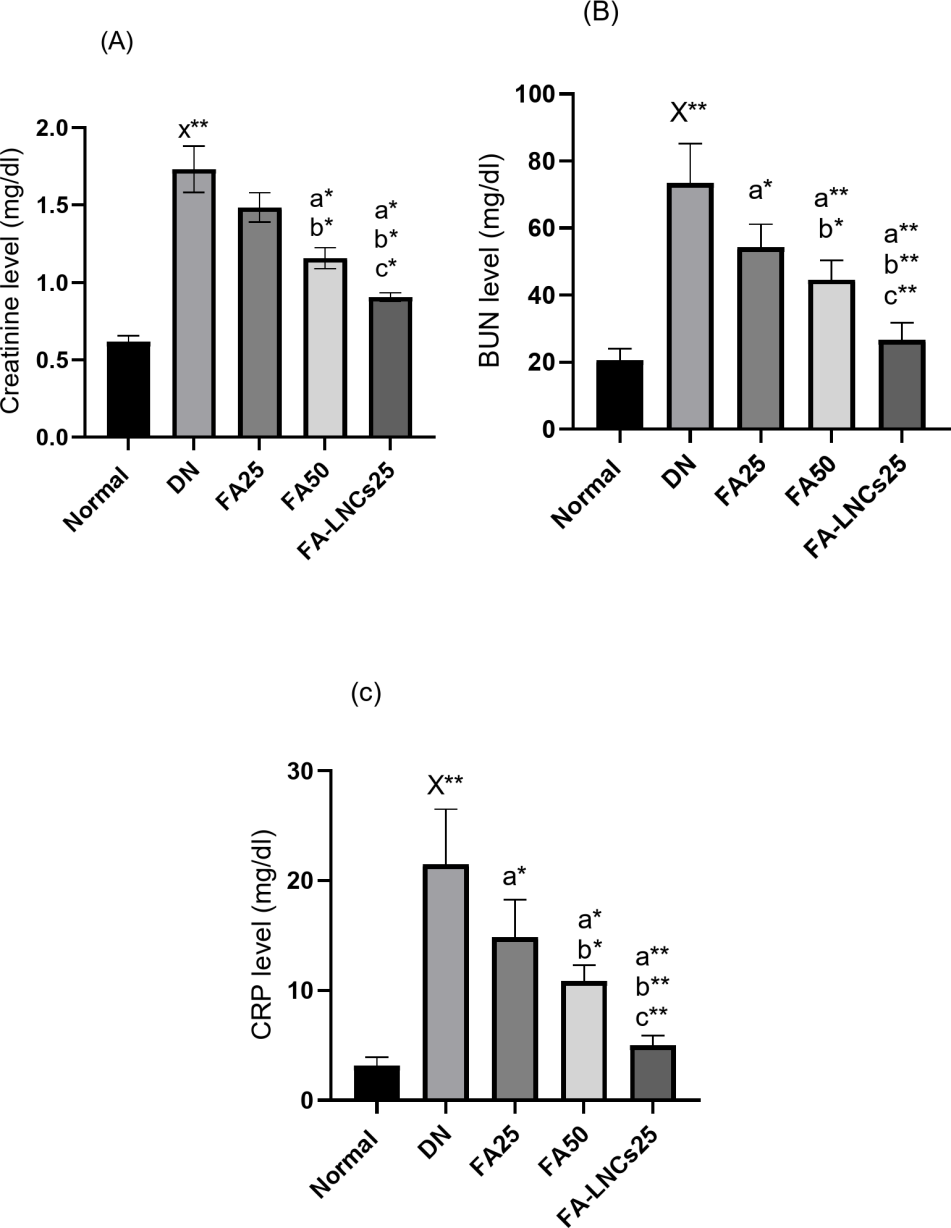

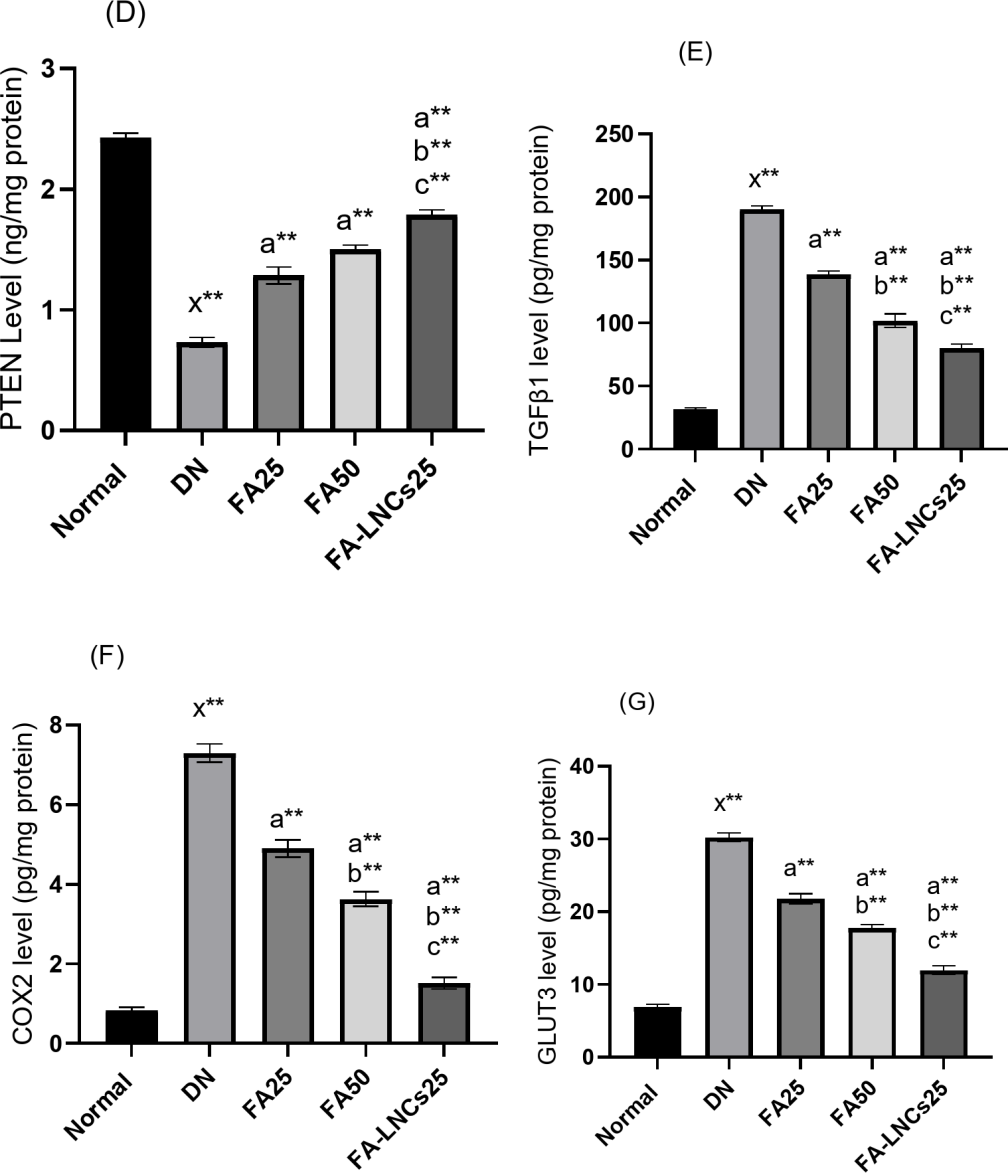
Effect of ferulic acid 25 mg/kg/day (FA25), ferulic acid 50 mg/kg/day (FA50), and ferulic acid lipid nano-capsules 25 mg/kg/day (FA-LNCs25) on (**A**) Creatinine level in serum. (**B**) Blood urea nitrogen (BUN) in serum. (**C**) C reactive protein (CRP) level in serum. (**D**) Phosphatase and tensin homolog (PTEN) level in kidney tissue. (**E**) Transforming growth factor beta 1 (TGF-β1) level in kidney tissue. (**F**) Cyclooxygenase 2 (COX2) level in kidney tissue. (**G**) Glucose transporter 3 (GLUT3) level in kidney tissue. Values were represented as mean ± SD. Number of rats in each group (*n* = 6). x: significant compared to normal group. a: significant compared to DN group. b: significant compared to FA25 group. c: significant compared to FA50 group. *: significant at *p* < 0.05 and **: significant at *p* < 0.001.

Figure 5A shows significant elevation in creatinine level in DN group by 63.6% (*p* < 0.001) compared to normal group. FA50 and FA-LNCs25 groups show significant decrease of creatinine level by 29.5% and 47%, respectively (*p* < 0.05), compared to DN group. Also, FA50 and FA-LNCs25 groups show significant decrease of creatinine level by 20% and 40%, respectively (*p* < 0.05), compared to FA25 group. FA-LNCs25 group demonstrates significant decrease in creatinine level compared to FA50 group by 25% (*p* < 0.05).

### Effect of different treatments on blood urea nitrogen (BUN) level in serum

Figure 5B shows significant elevation in BUN level in DN group by 71.9% (*p* < 0.001) compared to normal group. FA25, FA50 and FA-LNCs25 groups show significant decrease of BUN level by 26.1% (*p* < 0.05), 39.4% (*p* < 0.001) and 63.7% (*p* < 0.001), respectively, compared to DN group. Also, FA50 and FA-LNCs25 groups show significant decrease of BUN level by 18.1% (*p* < 0.05) and 50.9% (*p* < 0.001), respectively, compared to FA25 group. FA-LNCs25 group demonstrates significant decrease in BUN level compared to FA50 group by 40% (*p* < 0.05).

### Effect of different treatments on C reactive protein (CRP) level in serum

Figure 5C shows significant elevation in CRP level in DN group by 85.1% (*p* < 0.001) compared to normal group. FA25, FA50 and FA-LNCs25 groups show significant decrease of CRP level by 31.2% (*p* < 0.05), 49.8% (*p* < 0.05) and 76.8% (*p* < 0.001), respectively, compared to DN group. Also, FA50 and FA-LNCs25 groups show significant decrease of CRP level by 27% (*p* < 0.05) and 66.2% (*p* < 0.001), respectively, compared to FA25 group. FA-LNCs25 group demonstrates significant decrease in CRP level compared to FA50 group by 53.7% (*p* < 0.001).

### Effect of different treatments on phosphatase and tensin homolog (PTEN) level in kidney tissue

Figure 5D shows significant decrease in PTEN level in DN group compared to normal group by 70.8% (*p* < 0.001). FA25, FA50 and FA-LNCs25 groups demonstrate significant increase in PTEN level by 46.2%, 53.3% and 61.1%, respectively (*p* < 0.001), compared to DN group. FA-LNCs25 group illustrate significant increase in PTEN level compared to FA25 and FA50 groups by 27.8% and 16.7%, respectively (*p* < 0.001).

### Effect of different treatments on transforming growth factor beta 1 (TGF-β1) level in kidney tissue

Figure 5E demonstrates significant increase of TGF-β1 level in DN group compared to normal group by 83.5% (*p* < 0.001). FA25, FA50 and FA-LNCs25 groups demonstrate significant decrease of TGF-β1 level by 27%, 46.4% and 57.7%, respectively (*p* < 0.001), compared to DN group. FA50 and FA-LNCs25 groups show significant decrease of TGF-β1 level by 26.5% and 42%, respectively (*p* < 0.001), compared to FA25 group. FA-LNCs25 group show significant decrease of TGF-β1 level by 21.2% (*p* < 0.001) compared to FA50 group.

### Effect of different treatments on cyclooxygenase 2 (COX2) level in kidney tissue

Figure 5F indicates significant increase of COX2 level in DN group compared to normal group by 89% (*p* < 0.001). FA25, FA50 and FA-LNCs25 groups show significant decrease of COX2 level by 32.8%, 50.6% and 79.5%, respectively (*p* < 0.001), compared to DN group. FA50 and FA-LNCs25 groups show significant decrease of COX2 level by 26.5% and 69.4%, respectively (*p* < 0.001), compared to FA25 group. FA-LNCs25 group show significant decrease of COX2 level by 58.3% (*p* < 0.001) compared to FA50 group.

### Effect of different treatments on glucose transporter 3 (GLUT3) level in kidney tissue

Figure 5G illustrates significant increase of GLUT3 level in DN group compared to normal group by 77.2% (*p* < 0.001). FA25, FA50 and FA-LNCs25 groups show significant decrease of GLUT3 level by 28.1%, 41.4% and 60.6%, respectively (*p* < 0.001), compared to DN group. FA50 and FA-LNCs25 groups show significant decrease of GLUT3 level by 18.4% and 45.2%, respectively (*p* < 0.001), compared to FA25 group. FA-LNCs25 group shows significant decrease of GLUT3 level by 32.8% (*p* < 0.001) compared to FA50 group.

### Effect of different treatments on m-RNA expression of mammalian sterile 20 like protein kinase 1 (MST1) in kidney tissue

**Fig. 6:**
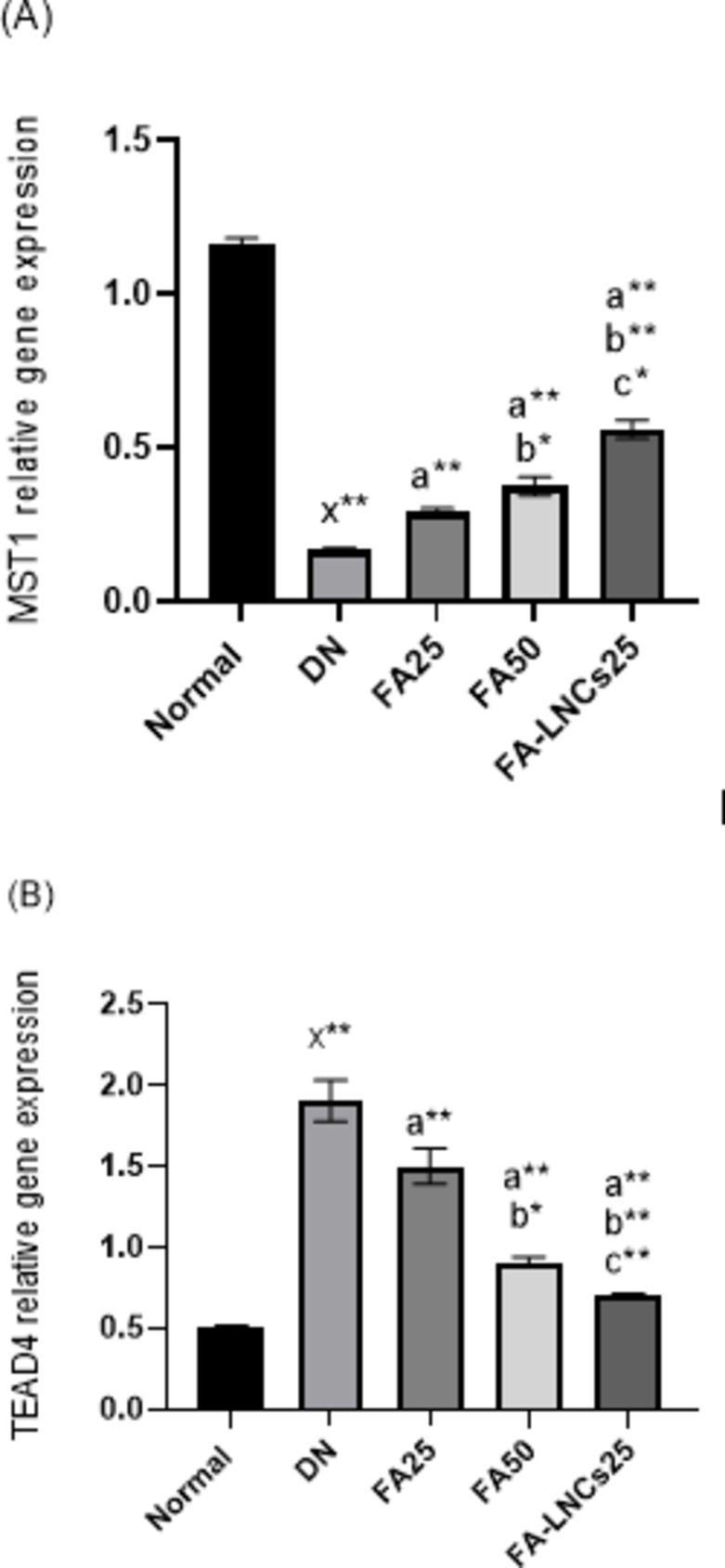
Effect of ferulic acid 25 mg/kg/day (FA25), ferulic acid 50 mg/kg/day (FA50), and ferulic acid lipid nano-capsules 25 mg/kg/day (FA-LNCs25) on (**A**) m-RNA expression of mammalian STE 20 like protein kinase 1 (MST1) in kidney tissue. (**B**) m-RNA expression of TEA domain transcription factor 4 (TEAD4) in kidney tissue. Values were represented as mean ± SD. Number of rats in each group (*n* = 6). x: significant compared to normal group. a: significant compared to DN group. b: significant compared to FA25 group. c: significant compared to FA50 group. *: significant at *p* < 0.05 and **: significant at *p* < 0.001.

Figure 6A illustrates significant decrease of MST1 relative gene expression in DN group compared to normal group by 85.3% (*p* < 0.001). FA25, FA50 and FA-LNCs25 groups demonstrate significant increase of MST1 relative gene expression compared to DN group by 41.4%, 55.3% and 69.6%, respectively (*p* < 0.001). FA50 and FA-LNCs25 groups show significant increase of MST1 relative gene expression compared to FA25 group by 23.7% (*p* < 0.05) and 48.2% (*p* < 0.001), respectively. FA-LNCs25 group shows significant increase of MST1 relative gene expression compared to FA50 group by 32.2% (*p* < 0.05).

### Effect of different treatments on m-RNA expression of TEA domain transcription factor 4 (TEAD4) in kidney tissue

Figure 6B shows significant elevation of TEAD4 relative gene expression in DN group compared to normal group by 72.6% (*p* < 0.001). FA25, FA50 and FA-LNCs25 groups show significant decrease of TEAD4 relative gene expression compared to DN group by 21%, 51.6% and 63.2%, respectively (*p* < 0.001). FA50 and FA-LNCs25 groups show significant decrease of TEAD4 relative gene expression compared to FA25 group by 38.7% (*p* < 0.05) and 53.3% (*p* < 0.001), respectively. FA-LNCs25 group shows significant decrease of TEAD4 relative gene expression compared to FA50 group by 23.9% (*p* < 0.001).

## Discussion

Diabetic nephropathy (DN) pathogenesis is complicated and multifactorial involving different pathways crosstalk^1^. The use of natural plant extracts with antioxidant and anti-inflammatory effects has drawn attention in its research field^18,19^. Ferulic acid (FA) has offered valuable effects on different disorders including kidney diseases^23,25^. Interestingly, FA is proved to have a significant antidiabetic action through modulation of hepatic glucose production, lipid metabolism and pancreatic cell function^45^. However, FA exhibits low oral bioavailability and very limited enteric permeability resulting in limiting its clinical application. So, inclusion of FA into nanotechnology-based formulation is the novel strategy to overcome its bioavailability drawbacks^27,28^. Accordingly, the present study is designed to investigate and compare the possible therapeutic effects of FA and its nano-form, ferulic acid lipid nano-capsules (FA-LNCs), on DN model in rats.

Streptozotocin (STZ) is used for induction of permanent pancreatic injury and generation of a rodent model of both type-1 and type-2 diabetes that develops renal injury with similarities to human DN. Research intended for examining the effects of treatments on this DN model should not start any treatments protocols until at least 3 weeks after STZ injection when kidneys have recovered from its acute nephrotoxic effect^29^. This comes in line with our preliminary study where kidneys of the PS4 group show dilated Bowman’s capsule, tubular dilation with cast formation in cortex, tubular dilation with epithelial cells degeneration and cast formation in medulla. This is considerably recovered in the kidneys of the PS6 group and progressively reappears again in PS8 and PS10 groups. Henceforth, the main study is designed to start the treatment protocol 10 weeks after STZ injection to eliminate any possible contradictory nephrotoxic effects of STZ and to ensure that the resulted nephropathy is a consequent of prolonged hyperglycemia.

Prolonged hyperglycemia is a major pathological factor in renal damage. Hyperglycemia-induced mitochondrial dysfunction and accumulation of reactive oxygen species (ROS) can directly result in modulation of signaling pathways and dysregulation of different cytokines with consequent induction of apoptosis and disturbing kidney function^46,47^. This comes in agreement with our results where DN control group shows elevated level of MDA as a consequence of oxidative stress progression and reduced level of GSH indicating malfunctioning antioxidant machinery.

TGF-β1 is a pleiotropic cytokine produced by different types of cells and is found to be dysregulated in inflammatory and fibrotic disorders^16^. Interestingly, renal expression of TGF-β1 is increased under diabetic conditions which comply with our results where DN control group show elevated level of TGF-β1 in kidney tissue. This is attributed to ROS where emerging evidence indicates that ROS induces and activates TGF-β1^17,48,49^.

Moreover, PTEN, a tumor suppressor protein, is found to be reduced in various types of cancers and in DN^50–52^. This complies with our results where DN control group show decreased PTEN level in kidney. This is credited to TGF-β1 where it is found to inhibit *PTEN* gene expression and decrease it level^53^.

The Hippo pathway is the latest addition to the family of signaling pathways known to be involved in control of fundamental biological processes^4^ and its dysregulation is found to be implicated in cancer^7,8^ pulmonary^9^, cardiovascular^10^, neuronal^11^, pancreatic^12^ and renal disorders^13–15^. MST1 is a core phosphorylating enzyme in the Hippo pathway. Its gene expression is found to be downregulated in diabetic conditions resulting in progressive inflammation^54,55^. This comes in agreement with our results where DN control group shows down-regulation of *MST1* gene expression in kidney tissue.

Furthermore, *TEAD4* of the hippo pathway is found to be upregulated in DN control group. This is also attributed to the TGF-β1, where previous studies pointed out that both TGF-β1 and TEAD4 can induce and trigger each other’s gene expression and activity^56,57^.

This dual inhibitory action on the Hippo pathway results in amplified expression of different target genes. GLUT3 is a direct transcriptional target of the deactivated Hippo pathway^58,59^. GLUT3 is found normally in the glomeruli and renal tubules^60,61^ and is found to be implicated in renal cell carcinoma^62,63^ which is proved to be induced by prolonged hyperglycemia^64^. This comes in agreement with our results where DN group shows elevated level of GLUT3 in the kidney tissue.

Another target is COX2 where it has been proven to show enhanced gene expression and elevated protein levels upon Hippo pathway deactivation^65^. Also, it is associated with renal inflammation^66^. This complies with our results where DN group illustrate elevated level of COX2 in the kidney tissue compared to normal group.

These actions are confirmed histopathologically where kidney tissue of the DN control group shows diffuse severe tubular damage including dilation with epithelial lining hydropic degeneration and coagulative necrosis, above and beyond significant elevation of creatinine level in serum of DN group is detected.

Contrariwise, groups treated with different doses and forms of FA demonstrate improvement of the histopathological image and serum creatinine level in variable degrees. FA25, FA50 and FA-LNCs25 groups show significant reduction of MDA and significant elevation of GSH compared to DN control group. Previous studies confirm our results where FA has been reported to have an antioxidant effect as a consequence to its antidiabetic action through management of STZ induced hyperglycemia in rats resulting in free radical scavenging and lipid peroxidation prevention^20,67^.

Additionally, these groups show significant modulation in TGF-β1 and PTEN levels besides *MST1* and *TEAD4* genes expression. This is attributed to the formerly mentioned FA-induced management of oxidative stress and hyperglycemia. Also, these groups demonstrate a significant decline in COX2 and GLUT3 levels which is attributed to the reactivation of the Hippo pathway by FA.

Previous studies explored the pharmacokinetics of ferulic acid lipid nano capsules administered orally in rats. These researches proved significant improvement of oral bioavailability through enhanced absorption and prolonged circulation time compared to its free form^68,69^. Also, other studies inspected its action on ischemic neural injury^70^ and acute pancreatitis injury^71^ before and after incorporation into lipid based nano capsules at equivalent doses. In both experimental models, the improved action of the nano form of FA was credited to overcoming its poor oral bioavailability. This comes in agreement with our findings. Our in-vitro release study indicated sustained release of FA from the nano formulation over 8 h. Along with, the significant improvement of the investigated parameters and histopathological features compared to FA25 and FA50 groups, which can be considered as evidence about the improved pharmacokinetics properties of our dosage form.

Collectively, this study points out, for the first time, that FA has the ability to alleviate DN by reactivating the hippo pathway through TGF-β1/Hippo pathway crosstalk modulation which improves its pathogenesis, with superior action of the FA-LNCs25 equivalent to 25 mg/kg/day of FA over the 50 mg/kg/day FA native form.

## Electronic supplementary material

Below is the link to the electronic supplementary material.

Supplementary Material 1

## Data Availability

All data generated or analyzed during this study are included in this published article.

## Abbreviations

COX2: Cyclooxygenase 2
DLS: Dynamic light scattering
DN: Diabetic nephropathy
FA: Ferulic acid
FA-LNCs: Ferulic acid lipid nano-capsules
GLUT3: Glucose transporter 3
GSH: Glutathione
H&E: Hematoxylin and eosin
LATS1/2: Large tumor suppressor 1/2
MDA: Malondialdehyde
MST1/2: Mammalian sterile 20-like kinase 1/2
PS: Particle size
PTEN: Phosphatase and tensin homolog
PDI: Polydispersity index
ROS: Reactive oxygen species
STZ: Streptozotocin
TAZ: PDZ-binding motif
TEAD4: TEA domain transcription factor 4
TGF- β1: Transforming growth factor-β1
YAP1: Yes associated protein 1
ZP: Zeta potential
