# Supplementary material for: Ferulic acid lipid nano capsules versus its native form in alleviating diabetic nephropathy induced in rats through TGF-β1/Hippo pathway crosstalk modulation
Source: Sci Rep. 2025 Mar 31;15:10979. doi: 10.1038/s41598-024-81175-7 (PMC11958811; doi:10.1038/s41598-024-81175-7)
Supplement: Supplementary file 1 — Supplementary Material 1 [file 41598_2024_81175_MOESM1_ESM.pptx]

## Slide 1
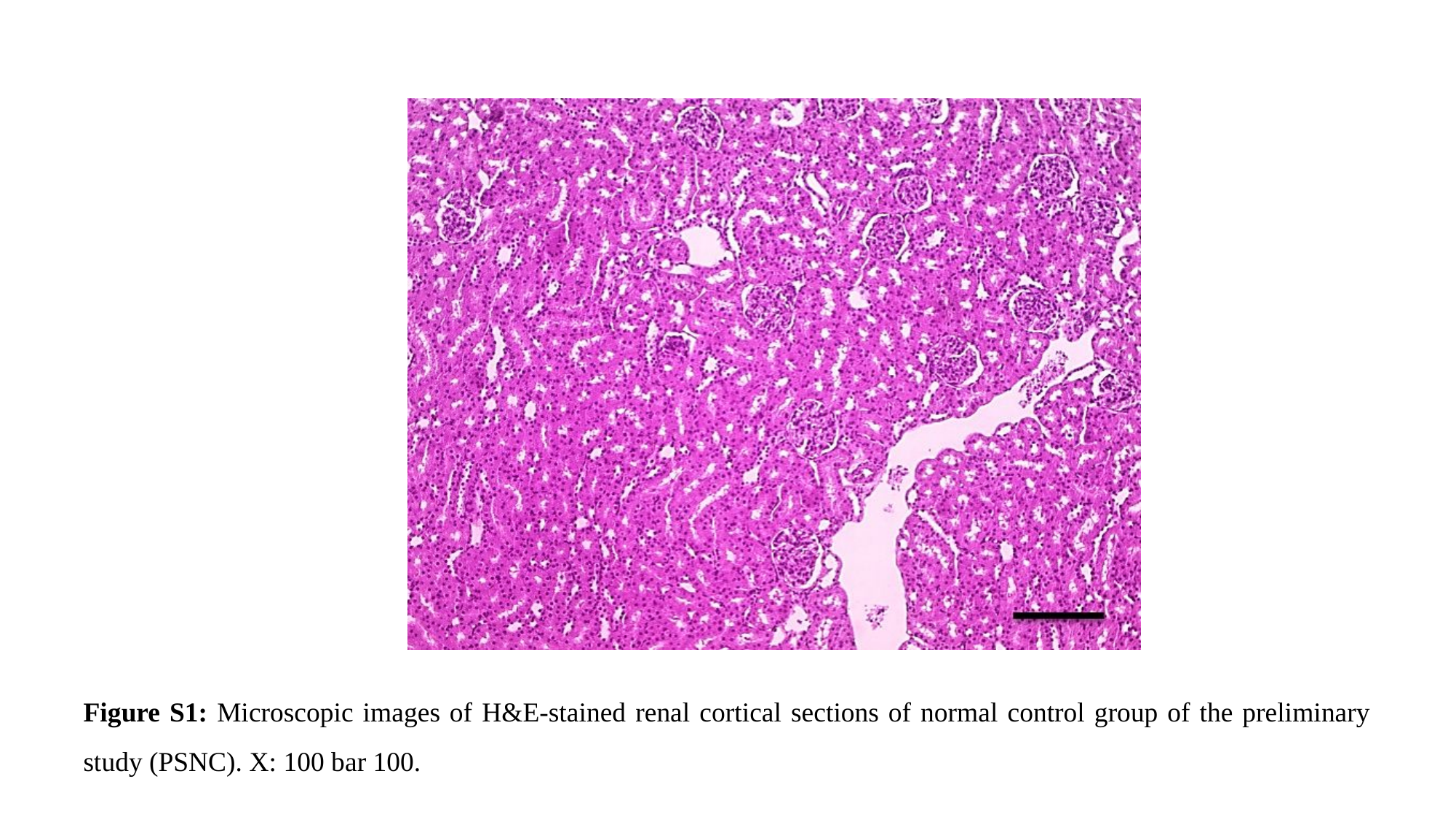

Figure S1: Microscopic images of H&E-stained renal cortical sections of normal control group of the preliminary study (PSNC). X: 100 bar 100.

## Slide 2
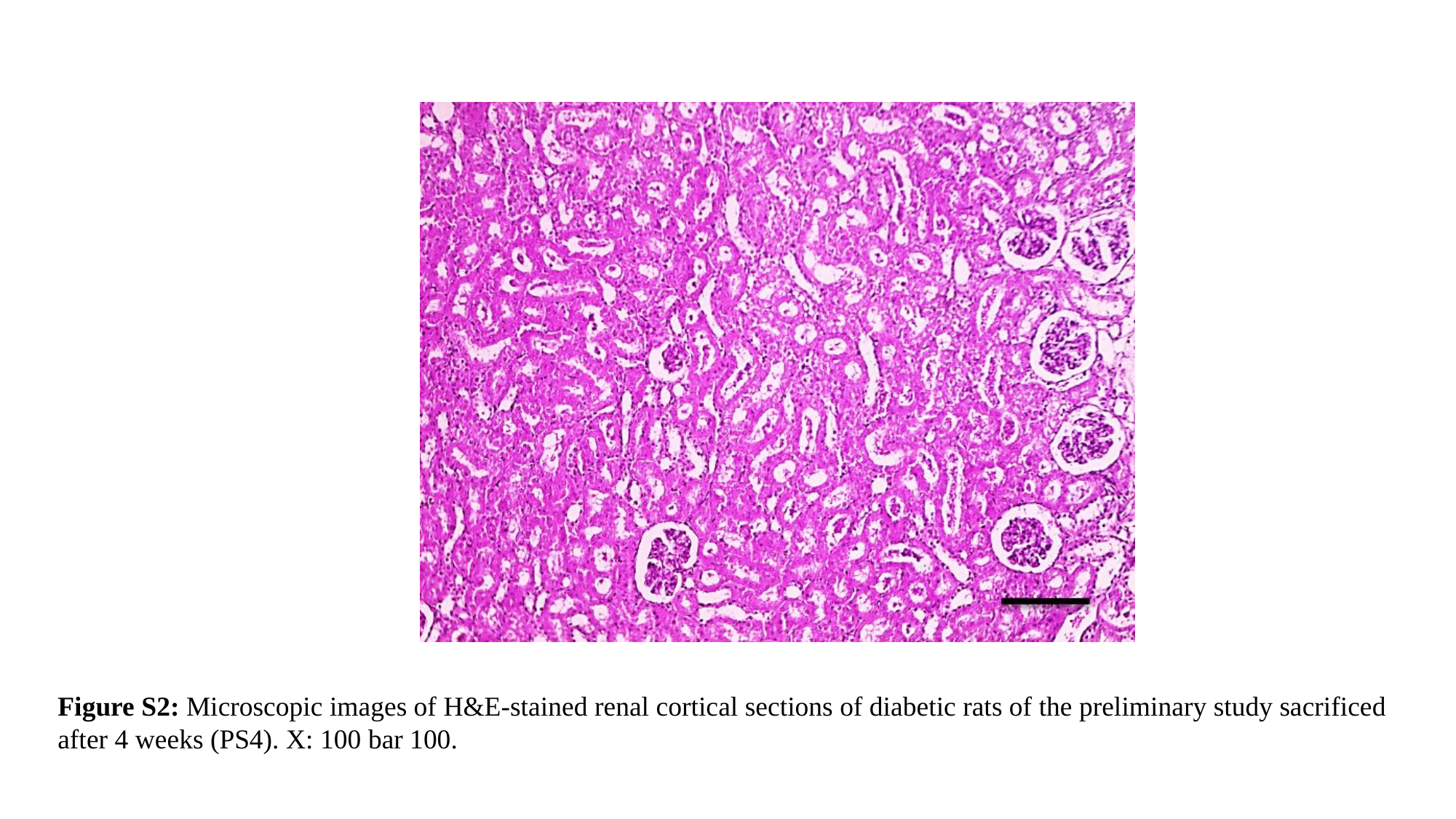

Figure S2: Microscopic images of H&E-stained renal cortical sections of diabetic rats of the preliminary study sacrificed after 4 weeks (PS4). X: 100 bar 100.

## Slide 3
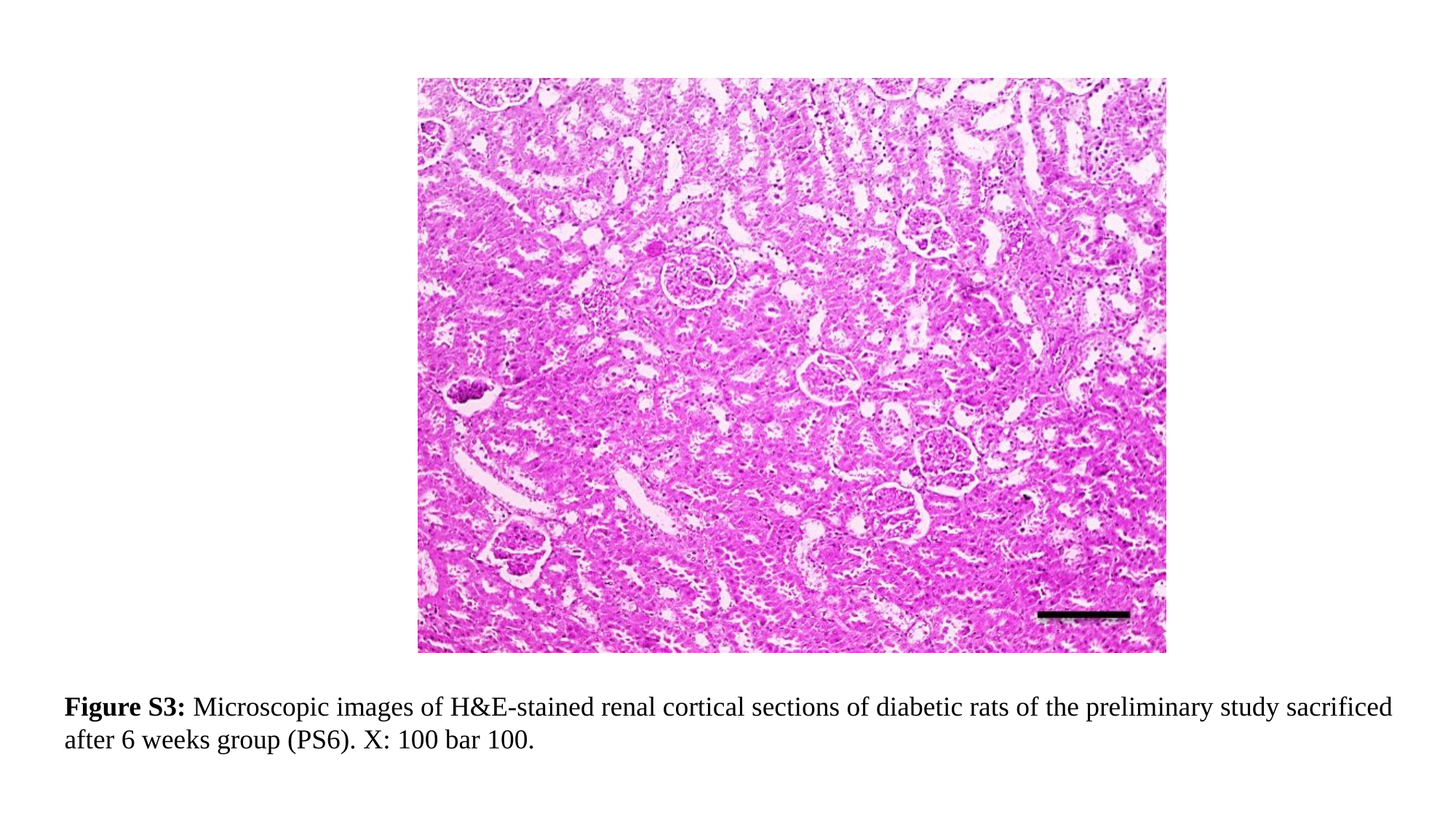

Figure S3: Microscopic images of H&E-stained renal cortical sections of diabetic rats of the preliminary study sacrificed after 6 weeks group (PS6). X: 100 bar 100.

## Slide 4
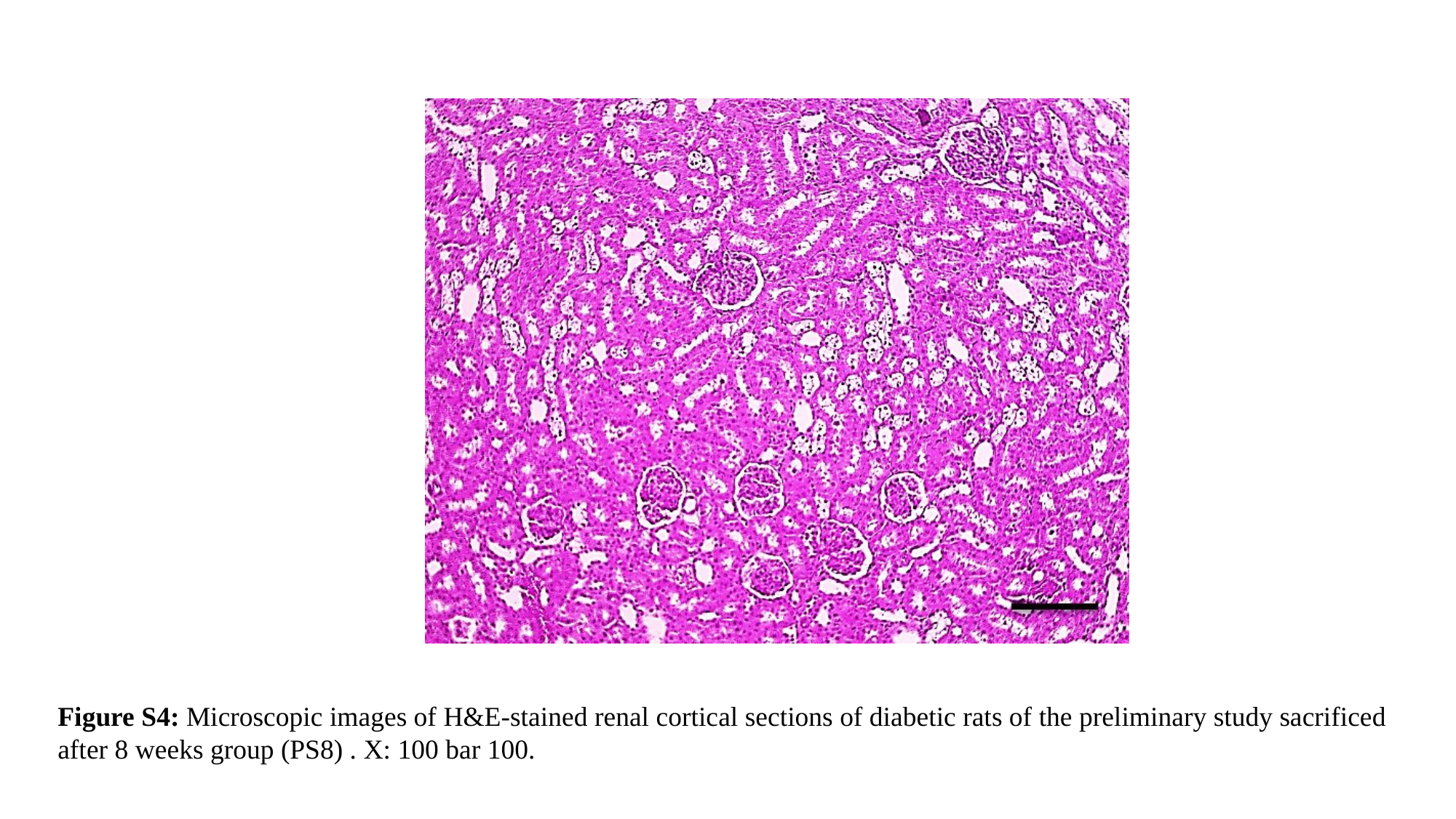

Figure S4: Microscopic images of H&E-stained renal cortical sections of diabetic rats of the preliminary study sacrificed after 8 weeks group (PS8) . X: 100 bar 100.

## Slide 5
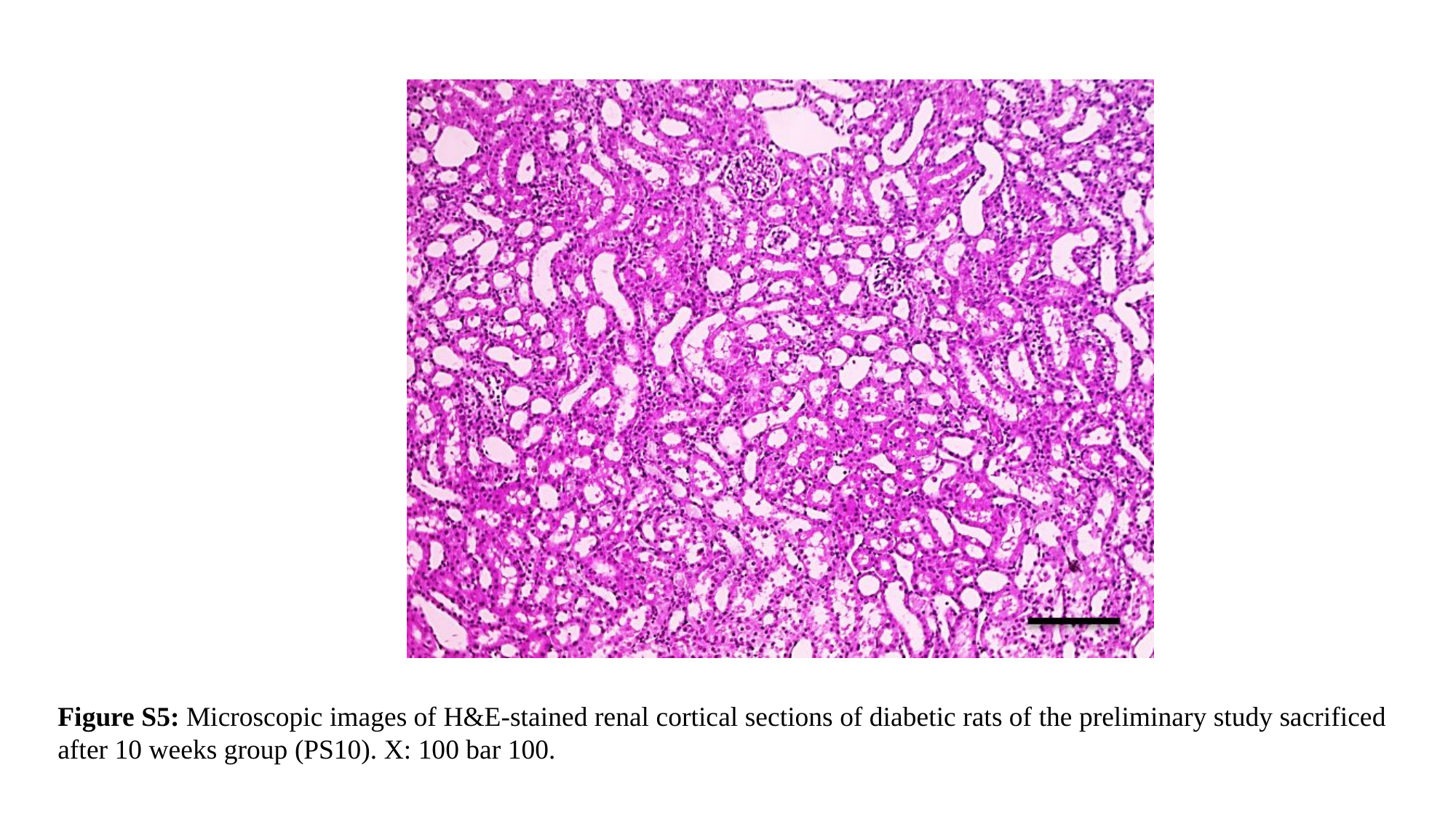

Figure S5: Microscopic images of H&E-stained renal cortical sections of diabetic rats of the preliminary study sacrificed after 10 weeks group (PS10). X: 100 bar 100.

## Slide 6
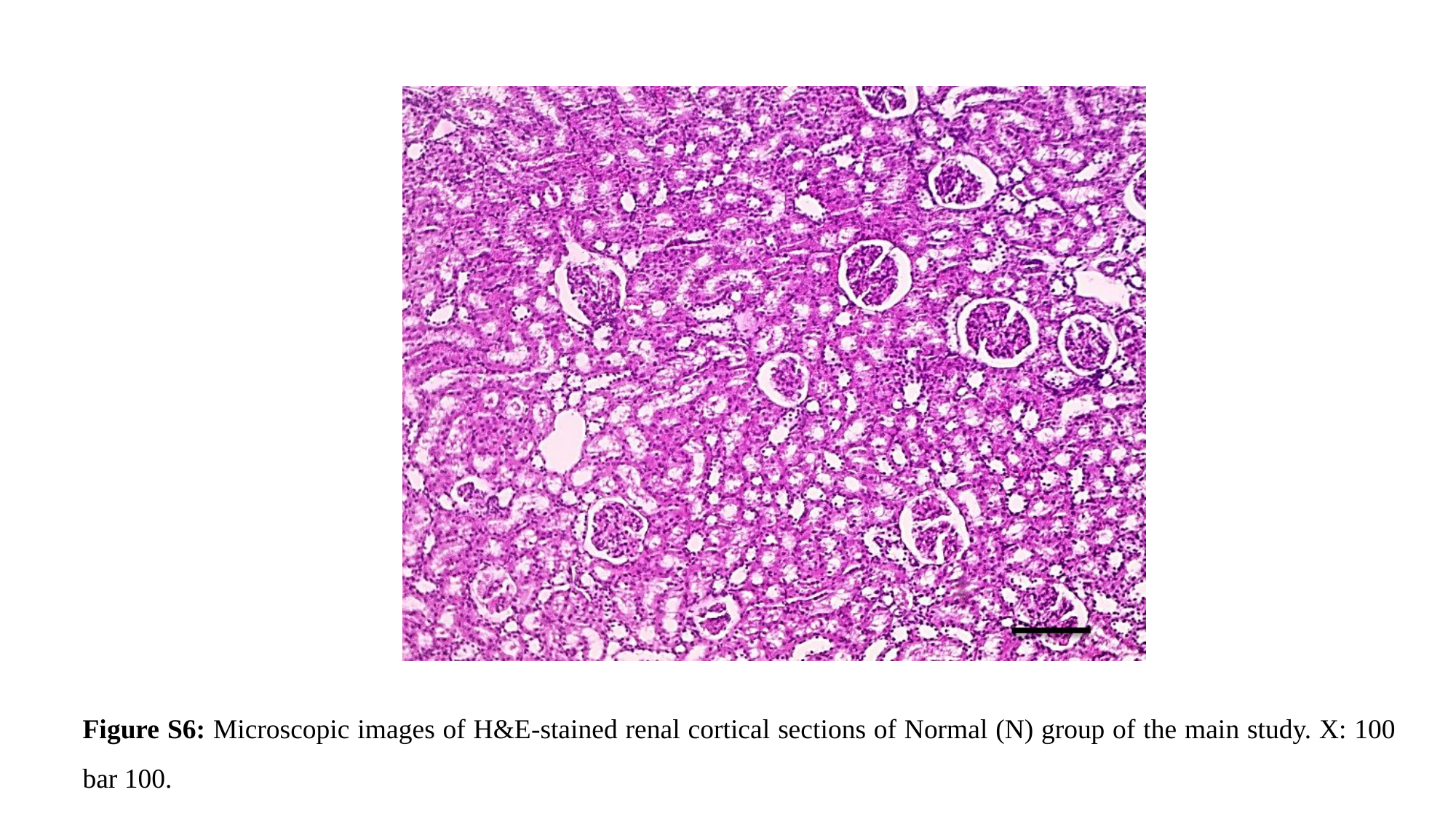

Figure S6: Microscopic images of H&E-stained renal cortical sections of Normal (N) group of the main study. X: 100 bar 100.

## Slide 7
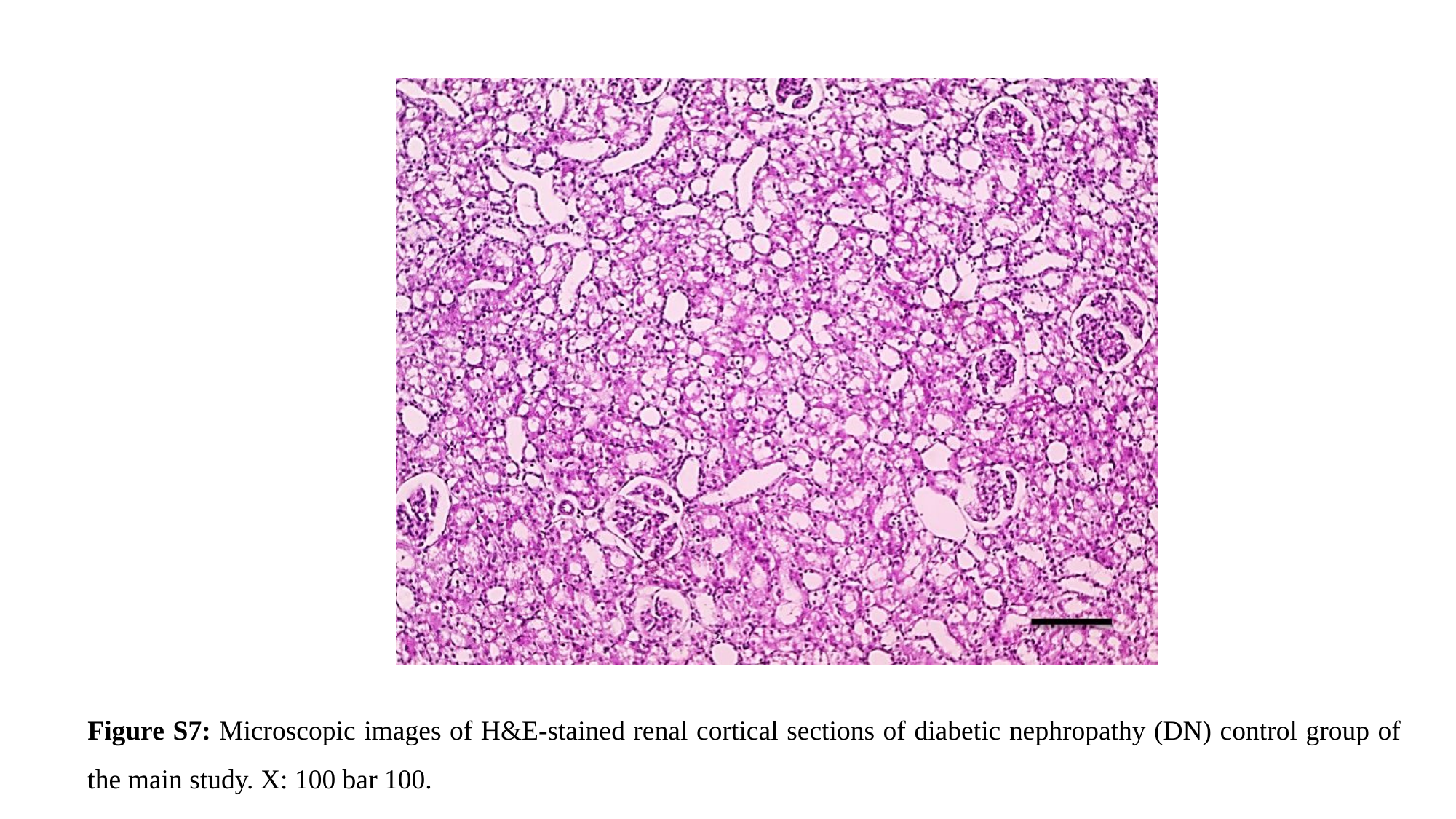

Figure S7: Microscopic images of H&E-stained renal cortical sections of diabetic nephropathy (DN) control group of the main study. X: 100 bar 100.

## Slide 8
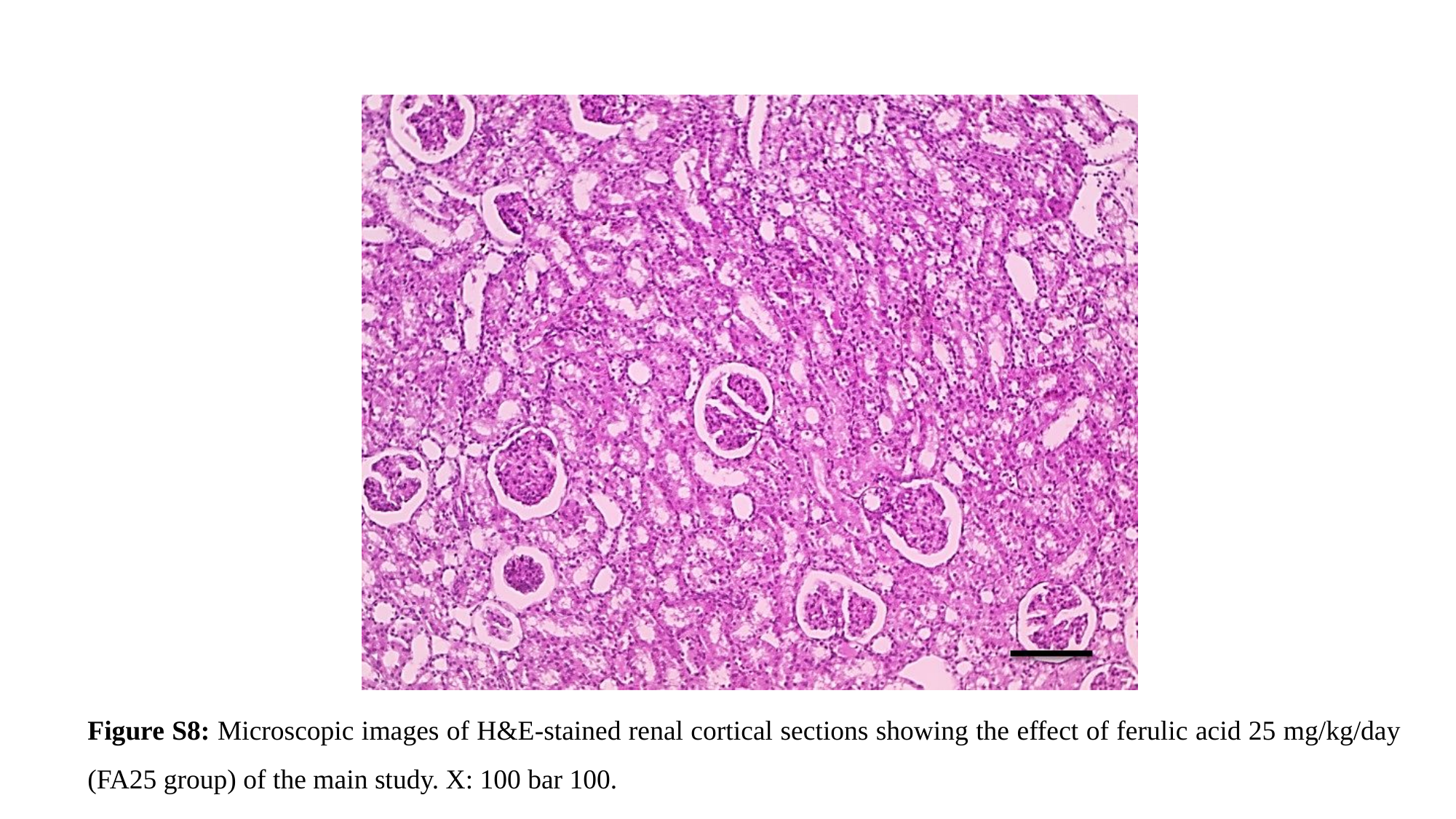

Figure S8: Microscopic images of H&E-stained renal cortical sections showing the effect of ferulic acid 25 mg/kg/day (FA25 group) of the main study. X: 100 bar 100.

## Slide 9
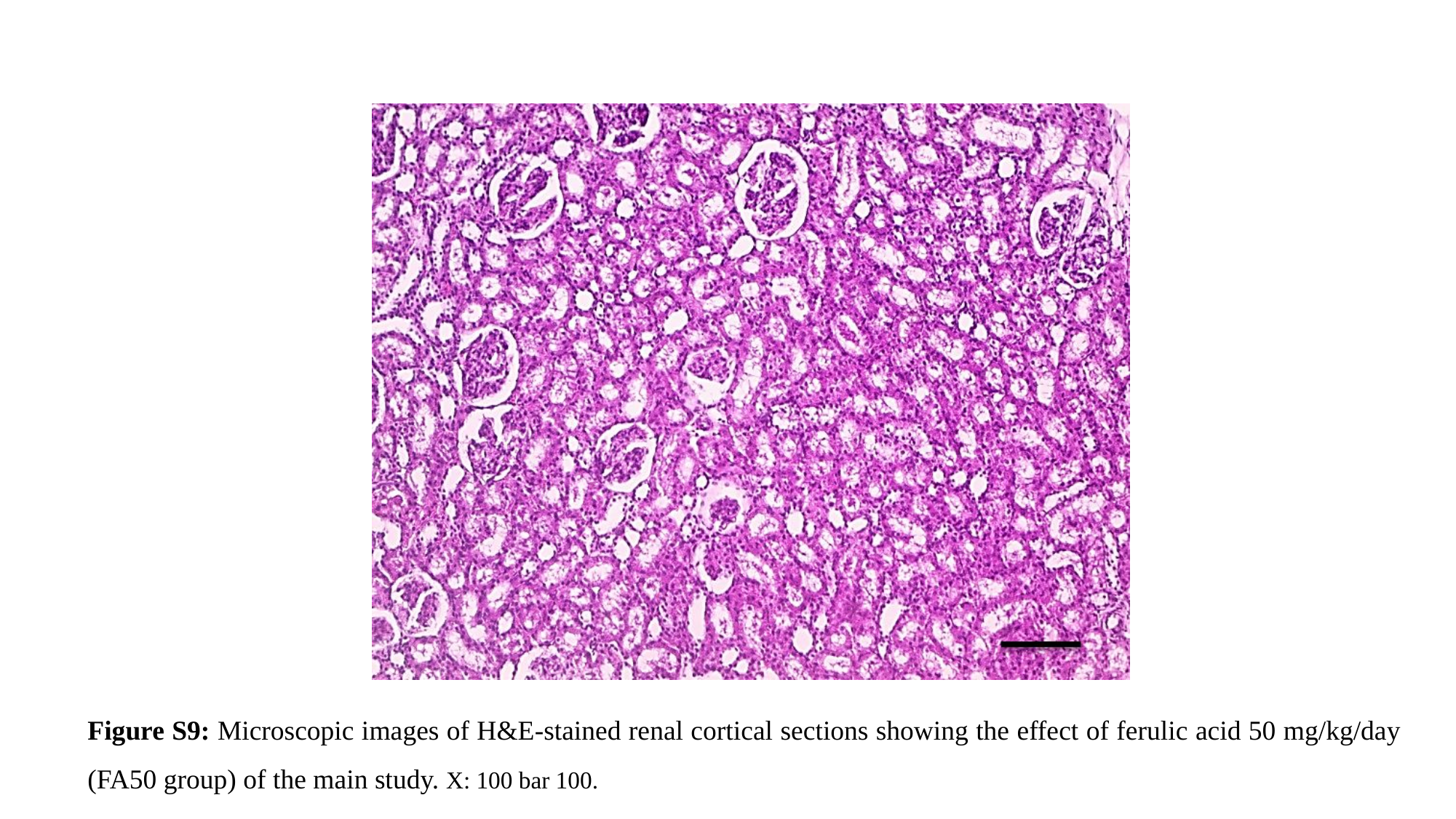

Figure S9: Microscopic images of H&E-stained renal cortical sections showing the effect of ferulic acid 50 mg/kg/day (FA50 group) of the main study. X: 100 bar 100.

## Slide 10
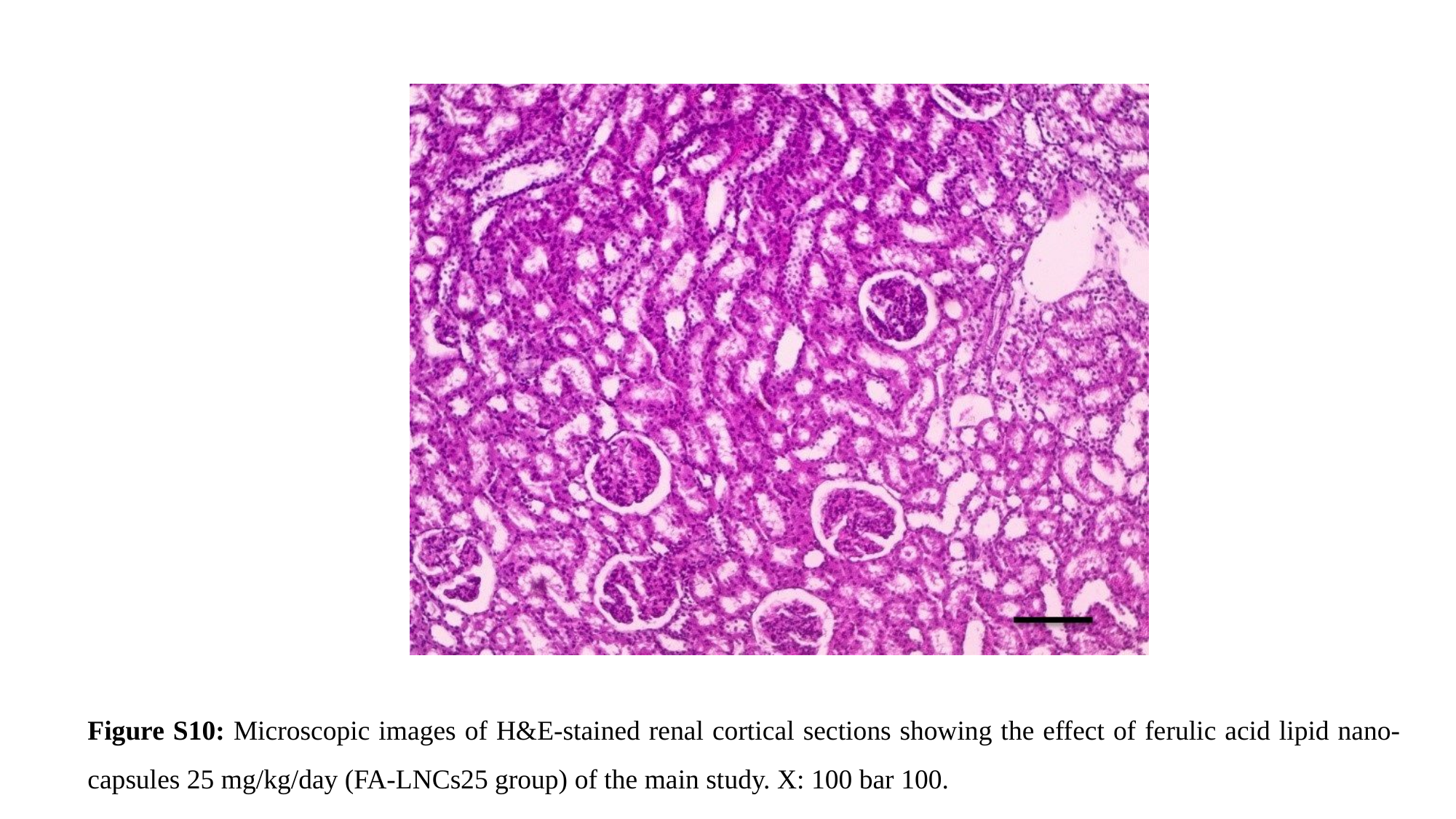

Figure S10: Microscopic images of H&E-stained renal cortical sections showing the effect of ferulic acid lipid nano-capsules 25 mg/kg/day (FA-LNCs25 group) of the main study. X: 100 bar 100.
